# Supplementary material for: Defining and measuring bedtime routines in families with young children—A DELPHI process for reaching wider consensus
Source: PLoS One. 2021 Feb 24;16(2):e0247490. doi: 10.1371/journal.pone.0247490 (PMC7904169; doi:10.1371/journal.pone.0247490)
Supplement: S1 Table — (DOCX) [file pone.0247490.s001.docx]

**S1 Table. Experts’ characteristics.**

|  | **Round I Expert group** | **Round II** | **Round III** | **Round IV** |
| --- | --- | --- | --- | --- |
| **N** | 11 | 25 | 20 | 13 |
| **Retention rate** | N/A | N/A | 80% | 65% |
| **Academic background**   - **Psychology** - **Public health** - **Dentistry** - **Education** - **Medicine** - **Policy** - **Nursing/midwifery/ health visiting** | 4  1  1  1  1  1  2 | 7  3  4  2  1  4  4 | 8  2  3  1  1  2  3 | 5  1  3  1  1  1  1 |
| **Professional area**   - **Research /academia** - **Clinical practice** - **Policy** | 8  1  2 | 15  4  6 | 15  2  3 | 10  1  2 |
| **For the expert group specifically** | | | | |
| **Chair** | **Prof Mike Kelly (University of Cambridge)** | | | |
| **Attendees** | Prof Jacqueline Barnes (Birkbeck University London)  Prof Iain Pretty (University of Manchester)  Dr Julia Allan (University of Aberdeen)  Dr Peter Day (University of Leeds)  Dr Robert Nettleton (Institute of Health Visiting)  Dr Anna Weighhall (University of Sheffield)  Mrs Fatima Hussain (Nat Cen Social Research)  Mrs Ellie Suggate-Francis (National Children’s Bureau)  Mrs Louise Mullins (City University London)  Dr Ming Wai Wan (University of Manchester) | | | |
