# Supplementary material for: Defining and measuring bedtime routines in families with young children—A DELPHI process for reaching wider consensus
Source: PLoS One. 2021 Feb 24;16(2):e0247490. doi: 10.1371/journal.pone.0247490 (PMC7904169; doi:10.1371/journal.pone.0247490)
Supplement: S2 Table — (DOCX) [file pone.0247490.s002.docx]

**S2 Table. Questions asked to experts as part of the DEPHI process.**

**Round II**

| Question | Answer | | | | |
| --- | --- | --- | --- | --- | --- |
| Before we start, can you please specify your job title and area of expertise/interest? All elements of this process are anonymised but knowing your job title and area of expertise/interest are important for our subsequent analyses. Be as specific and analytic as you like. |  | | | | |
| Definition of a good bedtime routine | | | | | |
| Below you will see the working definition of what constitutes a good bedtime routine as formulated by our expert group.  Please indicate whether you agree with the definition as presented or whether you want to make some changes/additions.  "A routine should be formed around a calm environment and include some key behaviours including brushing teeth, having a bath/shower, reading book/sharing a book or storytelling, singing, praying, avoiding stimulating activities like television/tablets-mobiles/video-gaming before bed and avoid snacks/drinks before bed. These activities should be fairly consistent over the week including the weekend. Finally, children should go to bed at a reasonable time each night depending on their age." | Agree with this definition | | | | |
|  | Agree, but some changes are necessary (use space below to specify changes) | | | | |
|  | Disagree, definition needs a major overhaul (use space below to specify changes) | | | | |
| Use this space to specify changes to the definition presented above. All proposed changes will be incorporated into the next stage of the process before reaching consensus. |  | | | | |
| Ranking of bedtime routine activities | | | | | |
| With bedtime routines encompassing a wide range of activities we would like to reach consensus on those activities which are essential for a good bedtime routine from a child health, wellbeing, development and parent wellbeing perspective. We recognise that all activities are important. However, some of them have more clear short and long-term implications for children and parents. When sharing our bedtime routines definition it will be good to highlight which of the activities included in bedtime routines definitely need to happen as frequently as possible to achieve best short and long-term results for children and parents alike. Also, a ranking of bedtime routines activities in terms of their priorities can help with future work in assessing and quantifying bedtime routines.  Please indicate which activities are most important and use the space provided at the end to add activities missing from the list. | Activity | Important to achieve  (*) | Very important to achieve (**) | Essential to achieve (***) |  |
|  | Toothbrushing |  |  |  |  |
|  | Book reading |  |  |  |  |
|  | Avoiding use of electronic devices |  |  |  |  |
|  | Avoiding snacks/drinks before bed |  |  |  |  |
|  | Singing, lullabies, praying, playing with children |  |  |  |  |
|  | Bath/shower before bed |  |  |  |  |
|  | Massage, cuddling, rocking |  |  |  |  |
|  | Consistency in getting to bed |  |  |  |  |
| Use this space to specify/add extra activities and their importance (using the three star ranking system). If you don't have any additions to make, then please use this space for comments on the ranking of bedtime routines activities. |  | | | | |

Round III

| Question | Answer | |
| --- | --- | --- |
| Before we start, can you please specify your job title and area of expertise/interest? All elements of this process are anonymised but knowing your job title and area of expertise/interest are important for our subsequent analyses. Be as specific and analytic as you like. |  | |
| Definition of a good bedtime routine | | |
| Below you will see the working definition of what constitutes a good bedtime routine as formulated by our expert group.  Please indicate whether you agree with the definition as presented or whether you want to make some changes/additions.  *“It is important to have a routine in place each night. A good bedtime routine can promote child health, development and wellbeing. Bedtime routines should be formed around a calm environment and include different activities such as: (1) brushing teeth right before going to bed (for children under 7, parents should actively brush children’s teeth), (2) avoiding snacks and drinks after brushing teeth & limiting snacks and drinks the hour before bed, (3) reading or sharing a book or telling a story before bed, (4) avoiding stimulating activities such as television, mobile phones, tables and gaming consoles, and (5) interacting with the child in calm, relaxing activities such as playing together, cuddling, singing and/or having a bath/shower but not necessarily every night. All these activities should take place during the hour before the child goes to bed and they should be fairly consistent across the week including the weekend. Finally, each night, children should go to bed early enough to allow them to sleep for the recommended, age-appropriate time before they have to get up in the morning and for a minimum of 8 hours each night.”* | Agree with this definition | |
|  | Agree, but some changes are necessary (use space below to specify changes) | |
| Use this space to specify changes to the definition presented above. All proposed changes will be incorporated into the next stage of the process before reaching consensus. |  | |
| Weighing of bedtime routine activities | | |
| You have 100 points to assign to each of the 6 activities listed on the table. These activities represent the most important and relevant activities that should form part of a family’s bedtime routine. In your professional opinion, assign your 100 points across these 6 activities keeping in mind the benefit(s) each activity can bring to children’s wellbeing and development. You can choose to equally distribute all your points or assign more/less to some activities depending on their importance. You have to use all of your 100 points. | Activity | Points awarded |
|  | Toothbrushing |  |
|  | Book reading |  |
|  | Avoiding use of electronic devices |  |
|  | Avoiding snacks/drinks before bed |  |
|  | Interactive activities with child before bed |  |
|  | Consistency in getting to bed each night |  |
|  |  | |
| Now, select one of the three weighing consistency options as listed on the table. You need to choose one of them or propose a new approach below. | Weighing option | Preference |
|  | (A) Multiple score by 1.0 if achieved 6-7 nights, 0.7 if achieved 4-5 nights, 0.5 if achieved 2-3 nights, 0.3 if achieved 1-2 nights and 0.1 if not achieved |  |
|  | (B) Multiple score by 1.0 if achieved every night, 0.9 if achieved 6 nights, 0.7 if achieved 5 nights, 0.5 if achieved 4 nights, 0.3 if achieved 3 nights, 0.1 if achieved 1-2 nights and 0.0 if not achieved |  |
|  | (C) Add each night’s scores and simply divide by 7 to achieve average score |  |
| Use this space to provide an alternative approach in weighing the consistency of the activities. |  | |

Round IV

| Question | | Answer | |
| --- | --- | --- | --- |
| Before we start, can you please specify your job title and area of expertise/interest? All elements of this process are anonymised but knowing your job title and area of expertise/interest are important for our subsequent analyses. Be as specific and analytic as you like. | |  | |
| Defining a good bedtime routine | | | |
| Provide the age range that you believe it’s appropriate for the bedtime routine definition we reached in round III to apply. You can choose any age range and you can make additional comments if you like. |  | | |
| Approach in assessing bedtime routines | | | |
| From the three options available (static, dynamic, both) which one do you prefer in terms of assessing bedtime routines? You can choose one of the approaches, both or neither. | Approach | | Preference |
|  | Static (one-off) | |  |
|  | Dynamic (repeated over a week) | |  |
|  | Both | |  |
|  | Neither | |  |
