# Supplementary material for: Defining and measuring bedtime routines in families with young children—A DELPHI process for reaching wider consensus
Source: PLoS One. 2021 Feb 24;16(2):e0247490. doi: 10.1371/journal.pone.0247490 (PMC7904169; doi:10.1371/journal.pone.0247490)
Supplement: S3 Table — (DOCX) [file pone.0247490.s003.docx]

**S3 Table. BTR-index.**

(a) Core activities to be covered when assessing bedtime routines in families with young children and scoring index for each activity

| **Activity** | **Achieved** | **Score** |
| --- | --- | --- |
| Brushing teeth before bed | YES/NO | If achieved, 35/100 |
| Time child goes to bed | YES/NO | If achieved, 20/100 |
| Reading/sharing a story before bed | YES/NO | If achieved, 15/100 |
| Avoiding food/drinks before bed | YES/NO | If achieved, 10/100 |
| Avoiding use of electronic devices before bed | YES/NO | If achieved, 10/100 |
| Calming activities before bed | YES/NO | If achieved, 10/100 |

(b) Types of assessment

| If assessing one-off, static bedtime routine scores (BTR-Index (S)) then participants will be provided with a score of 100 points if they achieved all 6 core activities described above.  This form of assessment focuses on bedtime routines the night before to minimise recall bias. |
| --- |

| If assessing bedtime routines over-time, dynamic measurement (BTR-Index (D)), then each activity should be scored for each night and then the weighing system should be applied depending on how many times the activity was achieved.  For a 7-night period, activity scores should be multiplied by 1.0 if they achieve the activity at least 6 (6-7) nights a week, 0.7 if they achieve the activity at least 4 (4-5) nights a week, 0.5 if they achieve the activity at least 2 (2-3) nights a week, 0.3 if they achieve the activity at least 1 night a week (1-2) nights and 0.1 if they don’t achieve the activity at all during the week. |
| --- |
