# Supplementary material for: Defining and measuring bedtime routines in families with young children—A DELPHI process for reaching wider consensus
Source: PLoS One. 2021 Feb 24;16(2):e0247490. doi: 10.1371/journal.pone.0247490 (PMC7904169; doi:10.1371/journal.pone.0247490)
Supplement: Attachment — Submitted filename: PONE-D-20-26667 Response to reviewers.docx [file pone.0247490.s004.docx]

**PONE-D-20-26667**

**Defining and measuring bedtime routines in families with young children; a DELPHI process for reaching wider consensus**

**RE: Response to reviewers**

Dear Goli,

First of all, on behalf of the authors, I would like to express our gratitude for the prompt review and consideration of our manuscript. We are glad that despite the suggested major revision of our manuscript the majority of the reviewers provided positive and supportive comments. We strongly believe that this manuscript has a lot of merit and it can provide an extremely useful insight in a widely common, repetitive family activity that can play a crucial role for children’s development and wellbeing.

Below you can find our detailed response to comments made by reviewers. We hope that our responses and subsequent actions, as mirrored in the revised manuscript, will be sufficient. We also took actions to reflect additional requirements listed in your email.

We look forward to hearing back from you.

Kind regards,

Dr George Kitsaras on behalf of the authors

| **Reviewer #1:** | |
| --- | --- |
| **Comment** | **Response & Action** |
| Did you carry out a structured review to present existing evidence to the expert panel in Round 1? If not, please address this limitation of not having done so and if you did, please include this detail in your manuscript. | This expert panel met in February 2020, 6 months into the start of our project (funded by MRC). The project overall is a follow-up of the PhD in Public Health examining bedtime routines and child wellbeing. During the PhD (and around a year and a half before the expert group met) there was a scoping and systematic review of the literature relating to the effects of bedtime routines interventions for child wellbeing and development (the results as not yet published). Combining results from the PhD overall, the scoping and systematic review and existing literature (see systematic review on bedtime routines and sleep by Mindell & Williamson 2018) we believe that there was sufficient evidence that was presented to experts during our February 2020 meeting. |
| It seems that you lost expert retention for subsequent rounds. Please address this address this limitation and what it means for your results. | We agree with this comment and to reflect this limitation we have included the following text on page 17 under “limitations”: *Also, there was an expert retention loss during the four round DELPHI process that could have led to some alternative voices and opinions missed from the final definition and proposed measurement. To counter lost retention, we provided experts with sufficient time to comment and provide feedback however, for a limited number of experts, that was not sufficient resulting in lost retention.”* |
| I am assuming you used 70% consensus agreement for all rounds? Is this correct? If not, please state what level you used for each round. And if you selected varying consensus levels for each round, please provide your reasoning as it is quite unconventional. If you did use 70% consensus, in Round 4, you accepted the proposed age range of 2-8 years, but you only have 8/13 = 62% | We apologise for this confusion. Yes, the 70% level of agreement was used throughout. For round IV we, mistakenly, omitted the fact that we used 2 attempts in trying to establish the age range of the proposed definition. In the 2^nd^ attempt, that produced the required level of agreement, the 2 experts that leaned towards the 1-7 and 1-8 age range revised their proposals and moved to the 2-8 age bracket. We have added the following text in page 8 to reflect this change: *“Due to a lack of 70% agreement in the first attempt, a subsequent, 2^nd^ attempt was necessary to reach the required level of agreement as with all other rounds.”* Table 1 has been also revised accordingly. |
| You propose a new index for measuring BTR from 0-100. Please clarify what your index scores indicates? (e.g., 0 = Poor; 100 = Excellent). Do you have a cut-off for "acceptable?" (e.g. 70)? | We have included a general statement on what the index scores indicate on page 12: *“(0% no routine in place, under 50% sub-optimal bedtime routine, 100% excellent bedtime routine)”* and we have added a sentence under “limitations” page 17 to reflect the need for additional work to fully explore and establish robust cut-off points: *“For the proposed index, further explorations will need to be in place moving forward to fully examine different cut-off points and determine where a routine seizes being beneficial and optimal and slips into a suboptimal, poor routine.”* |
| Can you clarify, did all 27 families completed both a static and dynamic questionnaire? I am thinking so, but I just wanted to make sure | Yes, all 27 families completed both assessments. |

| **Reviewer #2:** | |
| --- | --- |
| **Comment** | **Response & Action** |
| The children’s age range from 2 to 8 years is a wide variation considering the developmental milestones, for instance, preschool and school going children, sleep during daytime in preschool children hampering the sleep at night. | This is a great comment, and it was a source of discussion and arguments during the face-to-face expert group as well as during the remaining DELPHI rounds. Quite quickly, it was clear that anything under the age of 2 will be really tricky to define and put into a more wide-holistic definition (due to different approaches in feeding etc.). Recognising the need for our definition to cater to a wide range of ages we tried to use active and understanding language throughout the definition where we are not punishing parents if they don’t achieve the recommended activities while recognising that each family (and child) is different. Our experts argued that there are overlapping areas for children’s development up to the age of 8 (for example, assisted or supervised toothbrushing up to age 7 recommended in the UK) where a definition such as the one we proposed can be useful in providing parents with a core range of goals/recommendations while allowing sufficient space for individuality and agility, so the definition does not constrict parents and children. We discussed, and we are looking into, developing a 0-2 definition and in further expanding the current proposed definition with more specific targets and recommendations. However, we are extremely mindful of the multiple fronts that parents of young children have to tackle and therefore we wanted, for now at least, to be providing the “bigger picture” tools and skills to them. |
| “A previous study” mentioned in the validation of bedtime routine measurement section needs to be clarified. | The reference to the previous study has been added. |
| Minor grammatical errors such as incomplete first two sentences of methods in abstract section and so on. | We apologise for the incomplete sentences in our abstract, these are now correct. |
| Uniformity in citing the references as per the journal requirement and complete citation of reference number 8. | Reference 8 has been amended. |

| **Reviewer #3:** | |
| --- | --- |
| **Comment** | **Response & Action** |
| Abstract does not reflect the results of the manuscript. | The abstract has been updated to better reflect results.  *“Results; At the end of the four round DELPHI process and with a consistent 70% agreement level a holistic definition of bedtime routines for families with young children between the ages of 2 and 8 years is proposed. Additionally, two approaches in measuring bedtime routines, one static (one-off) and one dynamic (7-night period) are proposed following the end of the DELPHI process. A Bland-Altman difference plot was also calculated and visually examined showing agreement between the measurements that could allow them to be used interchangeably.”* |
| What does the 1 1 3 and 8 mean in "Table 1 of the results section (Round IV) | Table 1, Round IV results have been updated to better explain the numbers listed and how we reached the necessary level of agreement. |
| It will be good to mention what is meant by static and dynamic measurement in the section of "measurement of bedtime routines: two approaches with weighting of options" | Page 10 already provides a brief explanation of what static and dynamic refer to: *“on a one-off (static) and a 7-night (dynamic) measurement for bedtime routines”.*  We have now also added a clarification on the subtitle of this section. |
| Weighting consistency requires a bit more of explanation of how the scores are assigned | We added the following sentence on pages 12-13 to provide more information and two examples on how parents receive their scores as part of the assessment: *“If a parent achieves all 6 areas as part of his/her bedtime routine, then they will receive a score of 100%, if they omit one or more of the activities, they will lose those points resulting in a lower overall score (for example, if they omit book reading/sharing a story before bed (-15 points) + if they allow use of electronic devices (-10 points) then the overall score will be 75%).”* As for the consistency over the week, we believe that the current explanation provided with a specific and detailed example on page 15 is sufficient. If the reviewer requires a clearer example, we will be more than happy to provide that. |
| How are the points assigned to the 6 activities in the new approach. Is it based on previous studies or is it the authors description? | The 6 areas were assigned points during the 3^rd^ round of the DELPHI process where experts were provided with 100 points to “spend” on each of the activities (which in return they were prioritised and selected by experts in round II). It was up to each expert to decide how to spend their points depending on which activity they considered to be the most important (or to distribute their points equally across all activities if they believed that all of them were equally important). This was based on a line of thinking where things like brushing children’s teeth each night, for decades, has been on the forefront of public health messaging etc. and we know that lack of brushing can result in tooth decay and other oral health issues therefore, an activity like brushing teeth might be more important than others considering the wider health consequences. That was our line of thinking but experts were free to reflect their own thinking when assigning their points. As it turns out, most of them approached the weighing of the 6 activities in a similar manner. To date, there is no weighing bedtime routine measurement where different activities receive different weights to allow for a more in-depth exploration. Obviously, in practice and when, hopefully, this questionnaire will be more widely used and implemented we might need to reconsider and recalculate the proposed weighting. We have listed this potential (and current limitation) on page 17. |
| No information about the existing dataset used in the study. | We have added a reference to reflect the dataset we used for validating the proposed measurement. |
| How can we say that the validation is robust. To whom are the static and dynamic scores, as obtained from the new method, compared to? | We refrained from labelling our proposed measurement as robust, far from that, our measurement is a first step towards an ultimately better and more agile measurement for bedtime routines and the many different activities that take place during those routines. For that reason, we tried to be open and detail all possible limitations for this paper. As mentioned, for this work, we aimed to firstly, define bedtime routines for young children and on the back of that provide an initial exploration for a different approach in assessing bedtime routines within the same population. We still need comparison studies (against available, validated assessments like the Bedtime Routines Questionnaire) and further exploration of the proposed weighting approach to arrive at a robust measurement. However, our preliminary findings when we recalculated scores assigned to parents’ bedtime routines in a previous study using the static and dynamic weighted approach provide some initial reassurance that this approach might prove robust and useful in the long run. |
| Which additional variables have been used to establish this new BTR index? | The proposed index covered 6 areas/activities identified by the expert group as the most important-relevant to bedtime routines for young children. In round I (face-to-face meeting), experts were presented with a long list of activities from signing, praying, cuddling, bathing/showering before bed etc. where they were asked to prioritise the ones, they believed to be most important-relevant that led to a more condensed list of activities that ultimately led to the selection of the 6 most important and relevant. The following sentence has been added on page 10 to reflect this step: *“some initial screening of the list was undertaken during the face-to-face meeting to condense the options before proceeding to the next DELPHI round.”* |

| **Reviewer #4:** | |
| --- | --- |
| **Comment** | **Response & Action** |
| The research has been done on an unexplored area, which is a great work. | We thank the reviewer for this comment, we strongly believe in the merit of our work and the need for this piece of research to be shared more widely. |

| **Additional requirements** | |
| --- | --- |
| **Comment** | **Response & Action** |
| Please ensure that your manuscript meets PLOS ONE's style requirements, including those for file naming | Title fonts have been updated, figure captions now appear in the appropriate places and there have been changes to reflect PLOS ONE style requirements.  We apologise in advance if we missed something, we will be more than happy to resolve any further issues as we move forward with our manuscript. |
| In your Methods section, please state whether you obtained consent from experts to publish their names prior to their engagement in this research. | The following sentence has been added: *“Experts provided consent in sharing their details as a requirement in participating in the expert group meeting.”* |
| Please include additional information regarding the survey or questionnaire used in the study and ensure that you have provided sufficient details that others could replicate the analyses. For instance, if you developed a questionnaire as part of this study and it is not under a copyright more restrictive than CC-BY, please include a copy, in both the original language and English, as Supporting Information. | We have provided a copy of the proposed questionnaire (S4) and we have now added an additional supplementary file (S3) with the questions asked to experts during the DELPHI process to allow for greater transparency. |
| Please amend either the abstract on the online submission form (via Edit Submission) or the abstract in the manuscript so that they are identical. | The abstract has been amended accordingly. |
| Please upload a copy of Figure 3, to which you refer in your text on page 14. If the figure is no longer to be included as part of the submission please remove all reference to it within the text. | We apologise but there is no fig.3, graph on fig.3. was previously combined with fig.2. since they presented similar findings. |
